# MXene‐Based Photothermal‐Responsive Injectable Hydrogel Microsphere Modulates Physicochemical Microenvironment to Alleviate Osteoarthritis

**DOI:** 10.1002/smmd.70006

**Published:** 2025-04-13

**Authors:** Zehua Gong, Linjie Chen, Xiaolei Zhou, Chunwu Zhang, Dražen Matičić, Dražen Vnuk, Zhifeng You, Linjin Li, Huaqiong Li

**Affiliations:** ^1^ Joint Research Centre on Medicine Xiangshan Hospital of Wenzhou Medical University Ningbo China; ^2^ Zhejiang Engineering Research Center for Tissue Repair Materials Wenzhou Institute University of Chinese Academy of Sciences Wenzhou China; ^3^ The Fifth Hospital of Jinhua Jinhua China; ^4^ Department of Orthopaedics Key Laboratory of Orthopaedics of Zhejiang Province The Second Affiliated Hospital and Yuying Children's Hospital of Wenzhou Medical University Wenzhou China; ^5^ Jiangxi Provincial Key Laboratory of Tissue Engineering School of Rehabilitation Medicine Gannan Medical University Ganzhou China; ^6^ Joint Centre of Translational Medicine The First Affiliated Hospital of Wenzhou Medical University Wenzhou China; ^7^ Clinic for Surgery, Orthopaedics and Ophthalmology Faculty of Veterinary Medicine University of Zagreb Zagreb Croatia; ^8^ Department of Urology The Third Clinical Institute Affiliated to Wenzhou Medical University, The Third Affiliated Hospital of Shanghai University Wenzhou People's Hospital Wenzhou China

**Keywords:** hydrogel microspheres, near‐infrared irradiation, osteoarthritis, photothermal‐responsive

## Abstract

Osteoarthritis (OA) is a physical lubrication microenvironment‐inadequate disease accompanied by a sustained chronic chemical inflammation microenvironment and the progression of articular cartilage destruction. Despite the promising OA treatment outcomes observed in the enhancement of lubrication inspired by ball bearings to reduce friction and support loads, the therapeutic effect of near‐infrared (NIR) irradiation‐based photothermal‐responsive controlled release “smart hydrogel microspheres” on OA remains unclear. Here, we prepared MXene/NIPIAM‐based photothermal‐responsive injectable hydrogel microspheres encapsulating diclofenac sodium using a microfluidic system. Consequently, NIR irradiation‐based photothermal‐responsive controlled release “smart hydrogel microspheres” demonstrate beneficial therapeutic effects in the treatment of OA by modulating the physical lubrication and chemical chronic inflammation microenvironment, laying the foundation for the application of smart hydrogel microsphere delivery systems loaded with bioactive factors (including agents, cells, and factors) to regulate multiple pathological microenvironments in regenerative medicine.

## Graphical abstract

Photothermo‐responsive hydrogel microspheres were synthesized using microfluidic technology, demonstrating controlled drug release under NIR laser irradiation and enhanced joint lubrication. The anti‐inflammatory efficacy, reduction in cartilage degradation, and effective management of osteoarthritis in rat models were confirmed, highlighting their therapeutic potential for clinical applications.

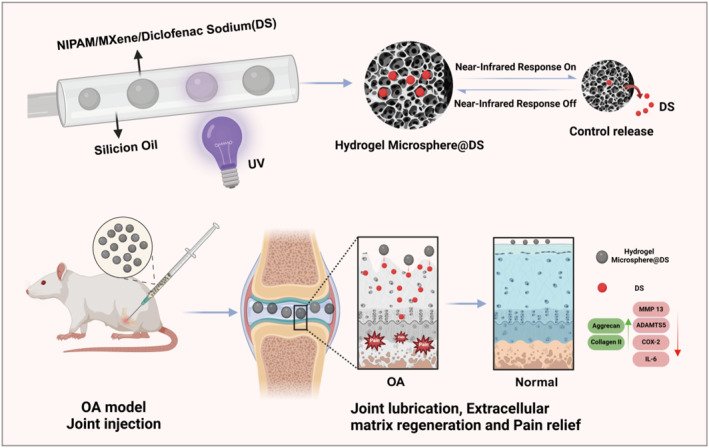

**Summary**

- MXene/NIPIAM‐based photothermal‐responsive hydrogel microspheres were fabricated.
- The microsphere system was developed for osteoarthritis (OA) treatment.
- The system could offer controlled drug release for anti‐inflammation.
- The design synergizes physical lubrication with photothermal effects.
- This work provides a multimodal strategy to mitigate OA symptoms.

## 1 Introduction

As a typical degenerative disease of the joint, osteoarthritis (OA) is characterized by structural and functional damage to the articular cartilage to result in pain, dysfunction, and disability [1, 2], posing a tremendous health and economic burden on an estimated 528 million [3] individuals worldwide in 2019. However, there is currently no cure for OA [4]. In consideration of the conventional clinical treatments for OA, surgery, a potential final alternative for OA by artificial joint replacement, may carry discomfort risk as a result of friction, and the patients probably suffer from pain and economic burden due to reoperation [5, 6]. Oral administration of special agents may result in adverse reactions in the body, such as gastrointestinal ulcers, owing to frequent dosing caused by poor uptake of agents into the cartilage [7, 8]. Instead, Intra‐articular drug injection treatment may reduce adverse reactions but at the cost of increased infection risk and decreased patient compliance due to frequent treatments [9, 10]. Moreover, these treatments fail to consider the crucial role of lubrication interventions in relieving OA symptoms, thereby highlighting the need for innovative therapies to improve lubrication and overcome frequent drug administrations.

Currently, it has been recognized that lubrication impairment resulting from mechanical and biological disruption that occurs upon cartilage damage is a key physical factor in triggering a cascade of pathological reactions, such as disruption of the cartilage matrix and chondrocyte behavior, thus aggravating OA [11, 12, 13], suggesting a potential optimal lubrication intervention to improve the outcome of OA patients. However, only a few approaches have been established to regulate the physical lubricant microenvironment. The injection of hyaluronic acid (HA)‐related hydrogel, a natural lubricant approved by the FDA [14, 15], has been demonstrated to provide enhanced lubrication ability within osteoarthritic joints [16]. However, this approach has also been associated with adverse effects, limited lubrication improvement, poor stability and unsatisfactory long‐term lubrication performance [17, 18].

Inspired by the function of spherical elements in ball bearings to reduce friction and support loads, hydrogel microspheres are now recognized as possessing significant potential for enhancing the lubrication of impaired joints [19]. Numerous studies have reported superior lubrication support compared to injected hydrogels, considering both the duration of support and the observed outcomes [20, 21]. However, the intervention of OA only in lubrication is not sufficient considering the complex microenvironment, including the chronic inflammatory response within OA, in inhibiting impaired cartilage regeneration.

Therefore, diclofenac sodium (DS), a non‐steroidal anti‐inflammatory drug (NSAID) used in the treatment of osteoarthritis to relieve symptoms such as inflammation, swelling, and joint pain [22, 23], was encapsulated in an injectable hydrogel microsphere with a uniform structure prepared by microfluidic channels in our group to modulate the physical lubrication microenvironment and chemical inflammation microenvironment simultaneously and to overcome the drawbacks of oral administration and intra‐articular drug injection in frequent dosing. Furthermore, in order to enhance the controlled release performance of DS within hydrogel microspheres, MXene, a 2D lamellar material used as a solid lubricant in tribology with excellent photothermal conversion efficiency (up to 48% under near‐infrared light irradiation at 808 nm) [24], and NIPIAM, a widely used thermosensitive material whose volume can be adjusted by temperature, were also incorporated into the hydrogel microspheres, thus achieving photothermally responsive controlled‐release drug delivery systems [25, 26].

**FIGURE 1:**
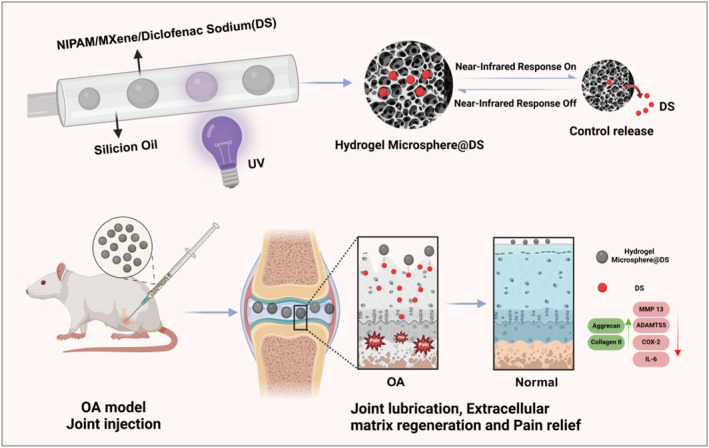
Schematic of the project. Hydrogel microspheres with adjustable diameters were prepared based on a microfluidic system, where the outer phase was attached to silicone oil, and the inner phase contained the precursor solution, including MXene/NIPIAM and diclofenac sodium (DS; drug treating osteoarthritis). Upon obtaining a uniform microsphere, the microsphere hydrogel was crosslinked by UV to establish NIR irradiation‐based photothermal‐responsive controlled release “smart hydrogel microspheres” and then injected into a rat osteoarthritis (OA) model to realize the beneficial therapeutic effect of OA by modulating the physical lubrication and chemical chronic inflammation microenvironment.

In this study, we conceived MXene/NIPIAM‐based photothermal‐responsive injectable hydrogel microspheres in which a dose of DS was loaded and controlled release by NIR irradiation to investigate the therapeutic effects of these delivery systems on OA by modulating the physical lubrication microenvironment and chemical inflammation microenvironment (Figure 1). The prepared hydrogel microspheres exhibited excellent characteristics such as uniform size (CV < 5%), solid photothermal response, and controlled release of the loaded agents. Furthermore, the injection of hydrogel microspheres encapsulated with DS in combination with NIR irradiation in an OA rat model exhibited restored joint space, improved collagen II/GAG deposition, and prolonged anti‐inflammatory effects. This work lays the foundation for further research on the application of NIR irradiation‐based photothermal‐responsive controlled release “smart hydrogel microspheres” for the treatment of multiple diseases in regenerative medicine.

## 2 Materials and Methods

### 2.1 Materials

The following materials were procured from Sigma‐Aldrich (USA) for use in this study: N‐isopropyl acrylamide (NIPAM, 97%), N, N′‐methylene‐bis (acrylamide) (BIS), poly (ethylene glycol)‐block‐(PEG‐PPG‐PEG, F108) and 2‐hydroxy‐2‐methyl‐1‐phenylacetone (HMPP). N‐methylol acrylamide (NMAM) was purchased from Aladdin Industrial Corporation (Shanghai, China). The Ti3C2Tx (MXene) thin‐layer dispersion solution was purchased from Nanjing XFNANO Materials Tech (Nanjing, China). Gelatin methacrylate (GelMA) was procured from Cure Gel (Wenzhou, China). Silicone oil (50 cs) was acquired from Shin‐Etsu Chemical (Shanghai, China). The Cell Counting Kit‐8 (CCK‐8, Enhanced) was purchased from Beyotime Biotechnology (Shanghai, China). Phosphate‐buffered saline (PBS), Dulbecco's modified Eagle's medium (DMEM), Nutrient Mixture F‐12 (DMEM/F12) culture media, fetal bovine serum (FBS), trypsin, and penicillin/streptomycin were purchased from Gibco (USA). Antibodies against matrix metalloproteinase (MMP 13), thrombospondin motifs 5 (ADAMTS 5), cyclooxygenase‐2 (COX‐2), Aggrecan, IL‐6, Collagen II, and IL‐1β were purchased from Abcam (USA). Radioimmunoprecipitation assay (RIPA) lysis solution was purchased from Thermo Fisher (USA). Phosphatase inhibitors, bicinchoninic acid (BCA) kits, and horseradish peroxidase (HRP)‐labeled goat anti‐rabbit IgG (H + L) were purchased from Beyotime (Shanghai, China). Polyvinylidene difluoride (PVDF) membranes were purchased from Bio‐Rad (USA). EDTA solution (10%) was obtained from Sangon Biotech (Shanghai, China). Diclofenac sodium (DS) was purchased from Solarbio Science & Technology Co. Ltd. (Beijing, China).

### 2.2 Establishment of the Microfluidic System

The microfluidic system is schematically illustrated in Figure 1. First, a capillary glass tube with an inner diameter of 580 μm was narrowed to a tip diameter of approximately 20–30 μm using a tube puller, and then the tapered end was polished to a final diameter of 70–80 μm using sandpaper, thus configuring it for use as an inner‐phase flow conduit. Furthermore, a similar capillary tube was employed for the outer‐phase flow device. The inner‐ and outer‐phase glasses were firmly anchored on the slide using AB powerful glue (Lantian, China) with yellow needles installed at each inlet to facilitate flow control.

### 2.3 Preparation of Hydrogel Microspheres

The outer phase of the flow device was connected to methyl silicone oil with a viscosity of 50 cs (the oil phase). The inner phase of the flow device was filled with an aqueous solution consisting of 10 wt. % NIPAM, 2.5% wt GelMA, 0.34% wt BIS, 0.025% wt MXene,  and 2.0% wt F108, with NMAM at the 10th of the NIPAM mass ratio, 1 vol. % HMPP, and 2 mg/mL DS. The mixture was thoroughly mixed using a magnetic stirrer and sonicated for approximately 30 min before the application. The solutions were propelled into the microfluidic system via polyethylene tubing, maintaining a flow rate of 5 mL/h for the outer phase and 0.8 mL/h for the inner phase. The internal phase syringe pump was first activated to ensure a stable flow before commencing the external phase pump. Once a stable droplet morphology was achieved, the resultant microcarriers were harvested into a centrifuge tube filled with deionized water post‐UV curing. Subsequently, multiple centrifugations were performed at 2500 rpm to eliminate the oil phase.

### 2.4 Characterization of the Microspheres Hydrogel

Diametric distribution: The diameters of the microsphere hydrogels were calculated using ImageJ (*n* = 50) images captured using an optical microscope (China, Nexcope, NIB620).

Scanning electron microscopy (SEM): A 70 μm cell sieve was used to remove the water used to disperse the hydrogel microspheres, leaving only microspheres. The microspheres were then rapidly frozen in liquid nitrogen and freeze‐dried in a freeze dryer. The dried microspheres were evenly spread on a table coated with conductive adhesive, followed by gold sputtering on their surface. The surface structures of the microspheres were imaged using SEM (Phenom Pharos, Phenom, Netherlands).

Lubrication Testing: The lubrication performance of PBS, hydrogel microspheres without MXene, and hydrogel microspheres with MXene were evaluated and compared using a UMT machine (Bruker, Germany). A stainless steel disc (Poisson's ratio: 0.3; elastic modulus: 194 GPa) was used as the bottom surface, while a UHMWPE ball (radius: 3.175 mm; Poisson's ratio: 0.46; elastic modulus: 0.7 GPa) was used as the top surface. The oscillation parameters were set to an amplitude of 4 mm, a frequency of 1 Hz, an applied force of 1 N, and a test duration of 300 s.

Photothermal properties: (i) Temperature variations were assessed under near‐infrared (NIR) irradiation (1.0 W/cm^2^) using a thermal imager across a range of MXene concentrations within the system (50, 100, 150, 200, 250, and 300 μg/mL). (ii) When the MXene concentration in the system reached 250 μg/mL, temperature variations were measured under varying power densities of NIR irradiation (0.5, 1.0, 1.5, 2.0 W/cm^2^). (iii) After irradiating the microspheres with NIR at a laser power of 2.0 W/cm^2^ until the Lower Critical Solution Temperature (LCST) was reached, NIR was deactivated to allow the microspheres to return to the ambient temperature. After a 30‐min interval, the microspheres underwent NIR irradiation once more. This procedure was repeated for 10 cycles, and the temperature fluctuations of the hydrogel microspheres were recorded. (iv) The NIPAM/MXene hybrid system was prepared as a circular hybrid hydrogel, and the temperature was set to 42°C to observe the color and morphological changes of the hybrid hydrogel. (v) Prepare a 10 wt% aqueous NIPAM/MXene polymer solution, and use differential scanning calorimetry (DSC, DSC 8000) to determine its LCST.

### 2.5 In Vitro Drug Release Experiment

To prepare NM@DS, DS was added to the previously described microsphere preparation solution to achieve a final concentration of 2 mg/mL. The prepared microspheres were divided into two groups and placed in centrifuge tubes containing 5 mL deionized water. The experimental group underwent NIR irradiation 20 times (5 times per day) while the control group remained untreated. After one NIR irradiation session in the experimental group, the supernatant was centrifuged (an equivalent volume of supernatant was taken from the control group), and the concentration of diclofenac sodium (DS) in the solution was determined using UV‐visible spectrophotometry (TU‐1901).

### 2.6 Isolation and Culture of Chondrocytes

3‐day‐old Sprague‐Dawley (SD) rats were obtained from the Zhejiang Provincial Laboratory Animal Center to isolate primary chondrocytes. All animal procedures in this study were approved by the Institutional Animal Care and Use Committee and were conducted following the governmental and the Wenzhou Institute, University of Chinese Academy of Sciences guidelines for animal welfare (approval NO. WIUCAS23020804).

Primary chondrocytes were isolated from a 3‐day‐old SD rat using collagen II digestion, according to the following protocol. Briefly, after anesthesia in rats, the cartilage was collected and segmented into granules by sterilized clipping, and the cartilage tissues were thoroughly cleaned with PBS three times before being transferred to a 15 mL centrifuge tube. The cartilage tissues were then digested with 1% type II collagenase (2 mg/mL) at 37°C for 4 h and centrifuged at 1000 rpm for 5 min to isolate the primary chondrocytes. The cell pellet at the bottom of the tube was resuspended in chondrocyte culture medium (10% fetal bovine serum and 1% penicillin‐streptomycin in DMEM/F12 medium) and seeded into cell culture flasks at 37°C in a 5% CO_2_ incubator. The culture medium was changed every alternate day. Once the primary chondrocytes reached 80%–90% confluence, they were subcultured.

### 2.7 Biocompatibility

The biocompatibility of the hydrogel microspheres was analyzed using an extraction method.

Briefly, microspheres from different groups were added to the primary chondrocyte complete culture medium at 0.2 g/mL for 3 days at 37°C to facilitate the leaching of potential cytotoxic compounds into the medium. The supernatant containing the substances extracted from the microsphere hydrogel was collected. The potential toxicity of the microsphere hydrogel due to MXene was first determined by collecting supernatant from the microsphere hydrogel with different concentrations of MXene ranging from 0 to 300 μg/mL. Chondrocytes at a density of 10^4^ cells/well in a 96‐well cell culture plate were treated with supernatants from different groups of microspheres, and cell viability was tested using the CCK8 kit following the manufacturer's instructions. Additionally, extracts from drug‐loaded microspheres, with or without NIR irradiation, were collected to assess the cytotoxicity of NIR irradiation using a CCK8 kit. The Live/Dead Cell Viability Assay Kit (Invitrogen, USA) was used to evaluate cellular viability, where primary chondrocytes were seeded at a density of 2.5 × 10^4^ cells/well in a 24‐well cell culture plate and treated with different kinds of supernatants for 5 days. On days 1, 3, and 5, cells in the different groups were stained with the kit and observed using an inverted fluorescence microscope (ZEISS, Germany).

### 2.8 Western Blot

**FIGURE 2:**
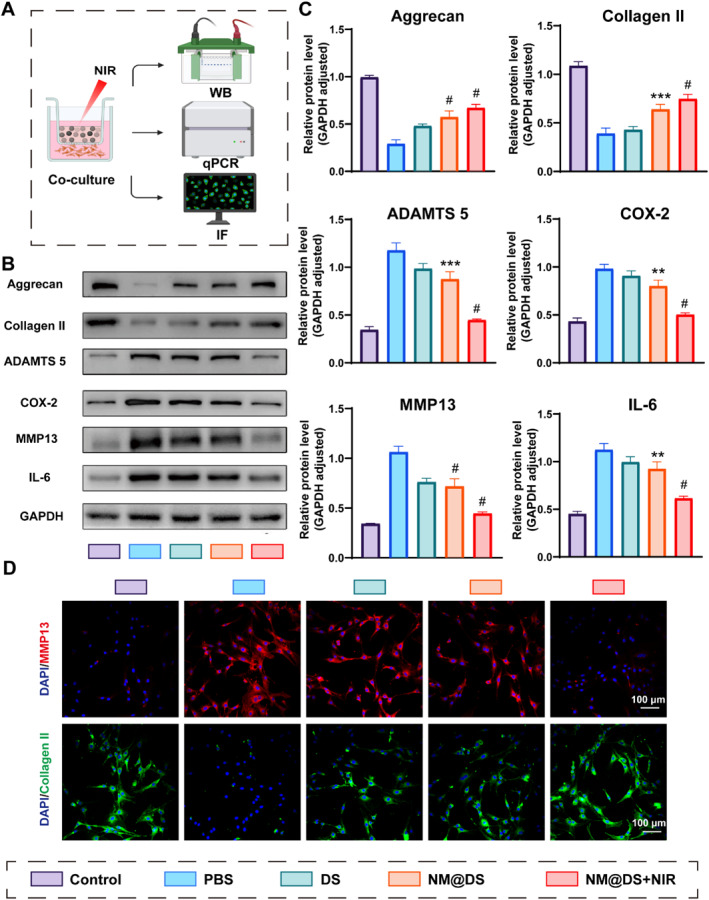
Beneficial effects of the NIR‐responsive release of DS‐loaded microsphere hydrogels on chondrocytes. (A) Schematic representation of the transwell system used in the test: microsphere hydrogels were plated on the insert, and chondrocytes were cultured on the bottom of the cell culture plate and tested by western blot, RT‐PCR, and immunofluorescent staining. (B) Western blot analysis of Aggrecan, Collagen II, MMP 13, ADAMTS 5; COX‐2, and IL‐6 levels in chondrocytes from each group. (C) Quantification of the relative protein levels of Aggrecan, Collagen II, MMP 13, ADAMTS 5, COX‐2, and IL‐6 according to the western blot results from B, *n* = 6. (D) Representative immunofluorescence images of MMP 13 (red) and collagen II (green) in chondrocytes from the different groups. Scale Bar, 100 μm (*n* = 3, **p < 0.01, ***p < 0.001, #*p* < 0.0001).

As shown in Figure 2A, after implantation of chondrocytes at a density of 5 × 10^5^ cells/well in a 6‐well cell culture plate, cells were incubated with IL‐1β (10 ng/mL) for 24h; during the incubation period, cells were treated with sodium diclofenac at a concentration of 2 mg/mL in the DS group or co‐cultured with DS‐encapsulated microspheres with or without near‐infrared laser irradiation for 20 cycles. The cells were then lysed in RIPA buffer containing a mixture of phosphatase and protease inhibitors. Following lysis, the lysates were centrifuged at 12,000 rpm for 30 min at 4°C to collect the supernatants. After testing the protein concentration with a BSA assay kit, the adjusted concentration of protein by 5 × Loading Buffer was denatured at 100°C for 10 min. 30 μg of total protein from each sample was loaded onto a sodium dodecyl sulfate‐polyacrylamide gel electrophoresis (SDS‐PAGE) system for separation, and proteins on the gel were transferred to a polyvinylidene fluoride (PVDF) membrane for immunoblotting. The membrane was incubated with specific primary antibodies overnight at 4°C to ensure optimal binding, followed by a 2‐h incubation at room temperature with horseradish peroxidase‐conjugated secondary antibodies. Subsequently, the membrane was washed three times with Tris‐buffered saline containing 0.1% Tween‐20 (TBST) to remove unbound antibodies. Chemiluminescence was detected and protein band intensities were analyzed using Image Lab software (version 3.0; Bio‐Rad Laboratories Inc.) and ImageJ software, which provided quantitative data on relative protein abundance.

### 2.9 qRT‐PCR Assay

Total RNA was isolated using the RNAiso reagent (TaKaRa, Japan). Subsequently, cDNA synthesis was performed using a RevertAid First Strand cDNA Synthesis Kit (TaKaRa) with 500 ng of RNA. Quantitative real‐time PCR (qRT‐PCR) was performed to amplify the cDNA using a SYBR Premix Ex Taq Kit (TaKaRa) and detected using a LightCycler 480 (Roche, China). mRNA levels of aggrecan, collagen type II, matrix metalloproteinase 13 (MMP13), Adamalysin‐like metalloproteinases with thrombospondin (TS) motifs (ADAMTS)‐5, aggrecan, and GAPDH were measured using specific primers and normalized against GAPDH expression. Primer sequences are detailed in Table S1 of the Supporting Information.

### 2.10 Immunofluorescent Staining

Chondrocytes cultured on cell culture plates were fixed with 4% paraformaldehyde for 15 min at room temperature, washed three times with PBS, permeabilized with 0.1% Triton X‐100 for 10 min, and blocked with 100 μL of 10% goat serum at 37°C for 1 h. Subsequently, excess fluid was removed and the cells were incubated with 100 μL of primary antibody at 4°C overnight, followed by a rewarming period of 1 h at 37°C, and then incubated with 100 μL of secondary antibody for 1 h at 37°C under light‐protected conditions. The nuclei were stained with DAPI. The slides were sealed with an anti‐fluorescence fading agent, and cellular fluorescence was assessed using laser confocal microscopy (Nikon A1).

### 2.11 Animal Model

Seven‐week‐old SD rats were adaptively housed for 7 days prior to the experimental procedures to establish rat osteoarthritis model. The rats were anesthetized and placed on the operating table to be clipped, and the skin around the right knee joint was antiseptically prepared to make an incision so as to separate subcutaneous tissues and sever the medial collateral ligament, thus extracting the medial meniscus from the joint cavity and lavishly irrigated with saline solution. Finally, the medial joint capsule and the initial skin incision were sutured. All animal procedures in this study were approved by the Institutional Animal Care and Use Committee and were conducted following the governmental and the Wenzhou Institute, University of Chinese Academy of Sciences guidelines for animal welfare. The following five experimental groups were compared: control, osteoarthritis (OA), diclofenac sodium (DS), microspheres encapsulating DS (NM@DS), and microsphere@DS with near‐infrared (NIR) light exposure (NM@DS + NIR). In the control group, neither the medial collateral ligament nor medial meniscus was excised.

Post‐surgical monitoring for any wound alterations was intensively performed, coupled with bi‐daily removal of rats from their housing to promote mobility, thereby facilitating osteoarthritis induction. Commencing 1 week subsequent to destabilization of the medial meniscus (DMM) surgery, the DS group received intra‐articular injections of diclofenac sodium into the right knee joint at weekly intervals; similar injection schedules were followed for the NM@DS and NM@DS + NIR groups, using hydrogel microspheres. Additionally, the NM@DS + NIR group was treated with near‐infrared laser irradiation bi‐daily (for four cycles each session). The OA group received saline injections within the knee joint and maintained the same injection frequency as the DS group. The control group was not subjected to any treatment.

### 2.12 Imaging Manifestations of Animal Knee Joints

Rats were anesthetized 4 or 8 weeks post‐surgery to collect the right knee joints with the knee capsules intact and fixed in 4% paraformaldehyde for 48 h. Radiographic analysis of the rat knee joints was performed using a digital X‐ray system (Philips Intera Achieva 3.0 T MR, USA). The imaging settings were optimized to an energy level of 50 kV and current of 160 μA. Subsequently, knee joint specimens were scanned and evaluated using a micro‐CT system (SkyScan 1272, Bruker). The obtained CT scans provided data for three‐dimensional reconstruction and further analysis using a dedicated software.

### 2.13 Histological Analysis

Knee tissue samples were fixed with 4% PFA and decalcified with 10% ethylenediaminetetraacetic acid (EDTA) in a shaker at 37°C for 2 months. The joints were embedded in paraffin and cut into 5 μm‐thick sections using a hard‐tissue slicer (Leica, RM2265). Hematoxylin and Eosin (H&E) and Safranin‐O Fast Green staining were performed as described previously [27, 28]. The Osteoarthritis Research Society International (OARSI) system was used to score histopathological changes in the sections, especially cartilage lesions and calcifications. In addition, immunohistochemical analyses targeting specific cartilage‐related proteins (Col2α,TNF‐α and MMP‐13) were performed.

### 2.14 Statistical Analysis

All assays were conducted in triplicates or more, and the raw data were statistically analyzed using GraphPad Prism version 8. Comparative assessments between various groups were performed using independent sample *t*‐tests and one‐way ANOVA, with outcomes reported as mean ± standard deviation. Notations for statistical significance are as follows: ns, no significant difference; *p < 0.05, **p < 0.01, ***p < 0.001, and #*p* < 0.0001.

## 3 Results and Discussion

### 3.1 Preparation and Characterization of Hydrogel Microspheres

**FIGURE 3:**
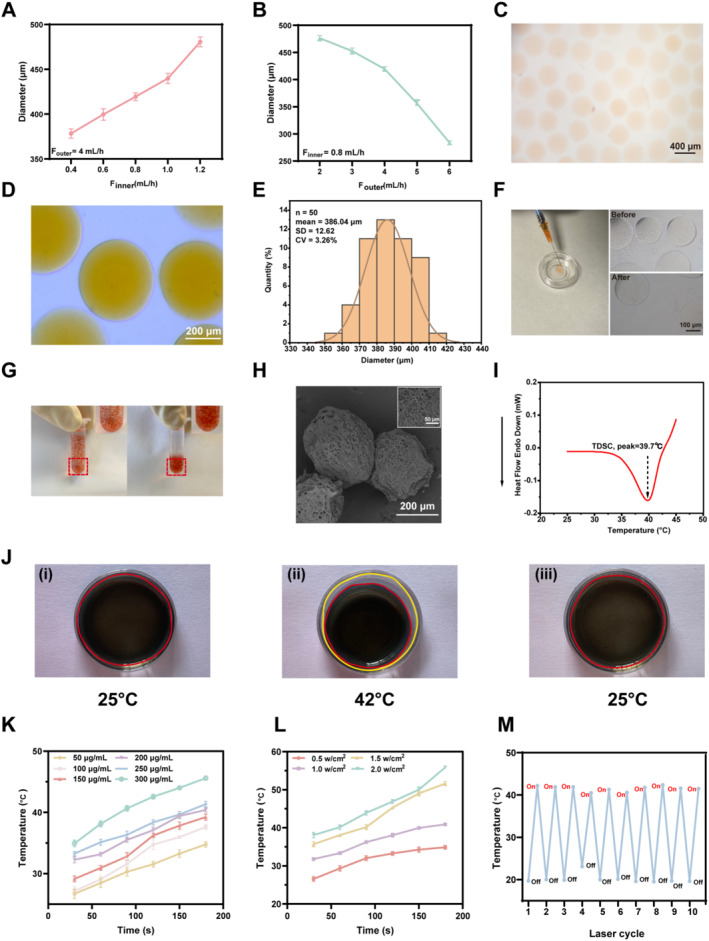
Preparation and characterization of near‐infrared (NIR) irradiation‐based photothermal‐responsive controlled release “smart hydrogel microspheres”. (A, B) Correlation analysis of the diameter of the prepared hydrogel microspheres with the flow rates of the external phase (A) and inner phase (B). (C) Representative images of the prepared hydrogel microspheres stained with azithromycin for easier observation. Scale bar, 400 μm. (D, E) Prepared hydrogel microspheres taken by an optical microscope (D) for diameter analysis (E), scale bar, 400 μm. (F) Representative morphologies of hydrogel microspheres with and without injection by syringe needle. (G) Precipitation analysis of hydrogel microspheres stained with azithromycin (left, rocking; right, approximately 10 minutes after rocking). (H) Representative images of hydrogel microspheres prepared using electron microscopy. (I) Differential scanning calorimetry (DSC) characterization of the LCST values of NIPIAM and NMAN solution. (J) Volume size change characterization of hydrogels placed on heated bench under different temperature conditions: (i) room temperature; (ii) 42°C; (iii) recovery from II to room temperature. The red circle highlights the hybrid hydrogel region, while the yellow circle indicates the container. (K) Temperature change of hydrogel microspheres containing different concentrations of MXene under NIR irradiation (1.0 W/cm^2^). (L) Temperature change of hydrogel microspheres with varying NIR irradiation powers (MXene: 250 μg/mL). (M) Temperature change in hydrogel microspheres during 10 cycles of NIR irradiation switching.

As a highly promising and widely used platform for biomedical purposes, hydrogel microspheres have been prepared using various methods, including microfluidics, mechanical crushing, emulsification, electrospraying, and so forth [29, 30]. In consideration of the superiority of microfluidics in terms of microsphere size uniformity [31], accurate and controllable encapsulation of drugs or cells [32], and batch‐to‐batch stability [33], the microfluidic platform established by our group [34] was used to prepare hydrogel microspheres (Figure 1). As expected, the diameters of the microspheres exhibited a dependence on the flow rates of the internal and external phases. An increase in the flow rate of the external phase (silicone oil) decreased the diameter of the microspheres (Figure 3A). Conversely, an increase in the flow rate of the internal phase, that is, the precursor solution, increased the diameter of the microspheres (Figure 3B). Consequently, microspheres were prepared utilizing an inner‐phase flow rate of 0.8 mL/h and an outer‐phase flow rate of 5 mL/h. Following the UV curing of the hydrogel microspheres, silicone oil from the external phase was eliminated through centrifugation, thereby facilitating the assessment of the microsphere morphology, size, and monodispersity using an optical microscope. Additionally, the prepared hydrogel microspheres were visualized in Figure 3C,D, exhibiting a precise uniform spherical morphology, which was stained with adriamycin hydrochloride to enhance the viability in the images.

Based on these images, the diameter of the prepared microspheres was calculated to be approximately 386.04 μm (Figure 3E), comfortably below the 450 μm diameter of a standard 1 mL syringe needle, ensuring good injectability, which was further verified that the hydrogel microspheres maintained a uniform and stable morphology after external injection through a 1 mL syringe needle (Figure 3F). Furthermore, the prepared hydrogel microspheres were uniformly dispersed in aqueous solutions upon gentle agitation to demonstrate their stability and ease of precipitation at room temperature, which are beneficial for injection. Moreover, the coefficient of variation (CV) of the diameter was 3.26%, which was below the 5% threshold, suggesting the excellent monodispersity of the microspheres in diameter (Figure 3E), stability, and ease of precipitation in aqueous solutions at room temperature (Figure 3G). Following lyophilization, the hydrogel microspheres were analyzed using scanning electron microscopy (SEM) to reveal the presence of a multitude of interconnected pore structures on the surface and within the microspheres, which facilitated the release of the agents loaded within the microspheres into the surrounding environment (Figure 3H).

The incorporation of NIPIAM enabled temperature‐sensitive characteristics of the prepared hydrogels, which have been previously reported to exhibit a lower critical solution temperature (LCST) of 32°C [35, 36]. At temperatures below the LCST, the polymer exists in aqueous solution because of the formation of hydrogen bonds between the hydrophilic groups and water molecules. Conversely, at temperatures above the LCST, the polymer undergoes a phase transition to form a hydrogel, accompanied by weakening of the hydrophilicity and enhancement of the hydrophobicity. Considering that the LCST of NIPIAM‐based hydrogels is not within the normal body temperature range, N‐methyl acrylamide (NMAM) was introduced into the hydrogel to adjust the LCST of the prepared hydrogels, as previously reported [37]. Differential scanning calorimetry analysis demonstrated that the adjusted LCST of the hydrogels was 39.7°C (Figure 3I), precisely within the normal body temperature range of 37°C–42°C, thus facilitating subsequent research on in vivo biomedical applications.

### 3.2 Photothermal and Lubricative Performance of Hydrogel Microspheres

Owing to the utilization of NIPAM and methacryloylated gelatin (GelMA) in the construction of temperature‐responsive hydrogels exhibiting the ability to undergo immediate deformation when exposed to temperatures above the LCST upon cooling, the hydrogels showed a reversible transition back to their original state. As illustrated in Figure 3J, when the hydrogels were positioned on a heated bench at 42°C, they exhibited visible crumpling accompanied by the extrusion of water, which was similar to a previous report, and we determined that the concentration of GelMA in the hydrogel was 2.5% [38]. The rapid change in the size of the hybridized hydrogel suggested good thermal responsiveness, which is essential for controlled drug delivery.

Furthermore, MXene, a material with numerous biomedical applications in fields such as cancer therapy, tissue engineering, and regenerative medicine, exhibits a remarkable photothermal conversion efficiency [39]. This phenomenon can be attributed to the intrinsic large absorption surface, abundant free electron distribution, and strong absorption across the broadband solar spectrum of the material [40]. Consequently, MXene exhibits an outstanding light energy absorption capacity and a distinctive localized surface plasmonic resonance (LSPR) effect, which enhances the conversion of light energy into thermal energy [41].

To examine the photothermal responsiveness, hydrogels containing varying concentrations of MXene were prepared to demonstrate that NIR laser irradiation increased the temperature of the hydrogels (Figure 3K). Furthermore, the duration of NIR laser irradiation was positively correlated with an increase in the temperature of the hydrogels, suggesting photothermal‐responsive characteristics. In addition, the higher the concentration of MXene in the hydrogel, the higher the temperature of the hydrogel at a constant duration of NIR laser irradiation. If the concentration of MXene in the hydrogels exceeded 250 μg/mL, the temperature of the hydrogels may be more than 42°C, which is harmful to cellular activity and results in tissue damage (Figure 3K). Moreover, a power response effect was observed with a fixed MXene concentration in the hydrogel, confirming the positive impact of the NIR laser power on the temperature of the hydrogel (Figure 3L). Consequently, 1.0 W/cm^2^ was determined for further analysis. Additionally, under irradiation with the NIR laser, the prepared hydrogel exhibited immediate photothermal responsiveness, showing an increase in temperature; however, when the irradiation was switched off, the temperature of the hydrogel returned to ambient temperature. This photothermal and repeatable responsiveness lasted for at least 10 on/off cycles (Figure 3M).

**FIGURE 4:**
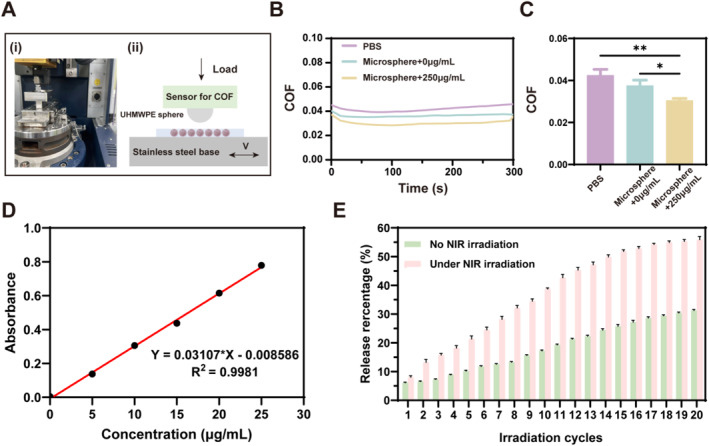
Lubrication and photothermal‐responsive controlled‐release performance of hydrogel microspheres. (A) Photograph (i) and schematic diagram (ii) of the UMT‐3 testing setup. (B) Coefficient of friction (COF) time curves for PBS, microspheres (MXene 0 μg/mL), and microspheres (MXene 250 μg/mL). (C) COF histogram comparing PBS, microspheres (MXene 0 μg/mL), and microspheres (MXene 250 μg/mL). (*n* = 3, *p < 0.05, **p < 0.01). (D) Standard curves for diclofenac sodium at different concentrations. (E) Cumulative release of DS from the hydrogel microspheres with and without repeated NIR irradiation (*n* = 3 per group).

The lubrication performance of prepared hydrogel microspheres in an aqueous solution was examined by a Universal Masterial Tester, as demonstrated in Figure 4A. The coefficients of friction (COF) in the phosphate‐buffered saline (PBS) group were significantly higher than those in microspheres without MXene and microspheres with MXene. In addition, the incorporation of MXene into the microspheres (microspheres with MXene) decreased the COF of the microspheres, confirming the lubrication of Mxene and excellent lubrication of the prepared hydrogel microspheres (Figure 4B,C).

### 3.3 Agent Loading and Release Capacities of Hydrogel Microspheres

To further assess the feasibility of NIR laser irradiation‐induced drug release, diclofenac sodium (DS)‐encapsulated within hydrogel microspheres was treated with NIR laser irradiation in subsequent experiments. In the absence of irradiation, the hydrogel microspheres demonstrated an initial agent release of 6.03%, which was calculated using an established standard curve correlating DS concentrations to absorbance at 276 nm (Figure 4D), reaching 30.09% after the sustained release of the agent. However, the hydrogel microspheres in the irradiation group demonstrated 7.85% release in the first cycle of irradiation. After 20 cycles of irradiation, the duration of which was equal to that of the hydrogel microspheres without irradiation group, the final release was 56.07%, approximately 1.8 times greater than that observed in the non‐irradiated group (Figure 4E).

Therefore, in the absence of NIR irradiation, the agent could only be released from the hydrogel microspheres at a markedly slow rate owing to the influence of diffusion forces. However, upon activation by NIR irradiation, the photothermal effect of MXene improves the microspheres and causes them to shrink, resulting in the accelerated release of the encapsulated agents. Following the NIR switch off, the microspheres cooled and returned to their initial state, preparing for the next triggered release.

### 3.4 Cell Biocompatibility and Beneficial Effect for IL‐1β‐Induced Chondrocytes

In order to evaluate the cell biocompatibility of the injectable photothermal‐responsive hydrogel microspheres, primary chondrocytes were isolated from newborn rats. Subsequently, a CCK8 assay was conducted to assess the effect of MXene concentration on the viability of the primary chondrocytes. Following a 24‐h incubation with extracts from hydrogel microspheres containing varying concentrations of MXene (0–300 μg/mL), the cells in each group exhibited comparable viability, indicating that MXene concentrations ranging from 0 to 300 μg/mL did not influence the biocompatibility of the hydrogel microspheres (Figure S1A). Moreover, the impact of near‐infrared (NIR) irradiation acting on injectable photothermal‐responsive hydrogel microspheres on primary chondrocytes was evaluated by CCK8 assay and Live‐dead staining. Primary chondrocytes were cultured in complete culture medium with or without NIR irradiation as well as extracts from DS‐encapsulated microsphere hydrogels with or without NIR irradiation for several days. The results demonstrated that there was no significant difference in cell viability among the various groups at different time points (Figure S1B). Furthermore, cell viability and number increased with an increase in culture time. A few dead cells were observed in each group, indicating that NIR irradiation and microspheres exerted a minimal impact on cell viability (Figure S1C). Similar results were observed for L929 cells (Figure S2). In summary, NIR‐irradiation‐based injectable photothermal‐responsive hydrogel microspheres displayed robust cell biocompatibility, making them suitable for use in biological applications.

Next, the effects of the hydrogel microspheres on osteoarthritis (OA) were investigated at the cellular level, as shown in Figure 2A, using a transwell system tested by western blot, Q‐PCR, and immunofluorescence staining. It has been reported that IL‐1β, one of the most important inflammatory factors accelerating the process of OA by activating multiple signaling pathways, is widely used as an important inducer to establish OA in vitro models at the cellular level [42, 43, 44]. After incubation with IL‐1β for 24 h, chondrocytes showed downregulation of aggrecan and collagen II at the protein level and gene expression, and upregulation of matrix metalloproteinase 13 (MMP 13) and thrombospondin motifs 5 (ADAMTS 5) (Figure 2B,C and Figure S3), which are critical enzymes involved in cartilage breakdown and play significant roles in the catabolic processes of the disease [45]. Additionally, cyclooxygenase‐2 (COX‐2), which plays an essential role in mediating prostaglandin synthesis and modulating pain, and IL‐6, a pro‐inflammatory cytokine, were also upregulated [46, 47], thus proving the accessibility of chondrocyte‐based OA cellular models induced by IL‐1β (Figure 2B,C). Diclofenac sodium (DS), a non‐steroidal anti‐inflammatory drug (NSAID) used to treat osteoarthritis to relieve symptoms such as inflammation, swelling, stiffness, and joint pain, has been reported to result in side effects of gastrointestinal bleeding with the use of an oral format [23], Therefore, topical distribution, including DS‐encapsulated microspheres (Microsphere@DS group) and NIR‐irradiation‐based injectable photothermal‐responsive hydrogel microsphere system (Microsphere@DS + NIR group), were evaluated to display superior therapeutic effects on the upregulation of aggrecan and collagen II, downregulation of MMP 13 and ADAMTS 5, and decreased secretion of IL‐6 and COX‐2, compared with the PBS‐treated group. In addition, the therapeutic effect was better in the Microsphere@DS + NIR group than in the DS‐treated‐and‐and Microsphere@DS‐treated groups (Figure 2B,C). Similar results for MMP‐13, ADAMTS‐5, aggrecan, and collagen II gene expression are illustrated in Figure S3 using quantitative real‐time PCR (qRT‐PCR), highlighting the enhanced therapeutic potential of NIR‐enhanced microspheres in modulating the molecular pathways involved in osteoarthritis (Figure S3).

To further elucidate the effect of the hydrogel microspheres on chondrocytes, immunofluorescence staining was used to assess key proteins, including Aggrecan, Collagen II, MMP 13 and IL‐6 (Figure 2D and Figure S4). Collagen II and Aggrecan levels were significantly decreased after IL‐1β induction in chondrocytes, whereas DS‐treated DS‐encapsulated microspheres without NIR irradiation increased collagen II levels. Furthermore, the DS‐encapsulated microspheres without NIR irradiation showed significant restoration of collagen II and Aggrecan fluorescence intensity, which was close to that of the control group (Figure 2D and Figure S4). However, for MMP‐13, a protein known to facilitate matrix degradation, the fluorescence intensity was strongly increased in the IL‐1β induction without treatment group. In contrast, MMP‐13 expression was downregulated after treatment with DS and DS‐encapsulated microspheres in the absence of NIR irradiation. Notably, the MMP‐13 fluorescence intensity levels in the DS‐encapsulated microspheres with NIR irradiation group were comparable to those in the control group, suggesting effective inhibition of matrix degradation (Figure 2D).

### 3.5 In Vivo Therapeutic Effect of Osteoarthritis

Before evaluating the in vivo therapeutic effect of hydrogel microspheres, the in vivo biocompatibility of DS‐loaded hydrogel microspheres with or without NIR irradiation was first assessed by HE staining of major organs, including the heart, liver, spleen, lungs, and kidneys, collected from OA rats after 8‐week treatment. The results demonstrated that cardiomyocytes, hepatocytes, and splenic and pulmonary tissues appeared structurally intact, exhibiting clear glomeruli, renal tubules, uniform cytoplasm, and no abnormal lesions, such as necrosis, edema, or inflammatory cell infiltration (Figure S5). In addition, the hematological parameters for alanine aminotransferase (ALT), aspartate aminotransferase (AST), blood urea nitrogen (BUN), and creatinine (CRE) tested by ELISA showed no significant differences between the injected microsphere group and the control group, suggesting that microsphere injection with or without NIR irradiation did not show any negative effects, with positive in vivo biocompatibility of the microspheres (Figure S5).

**FIGURE 5:**
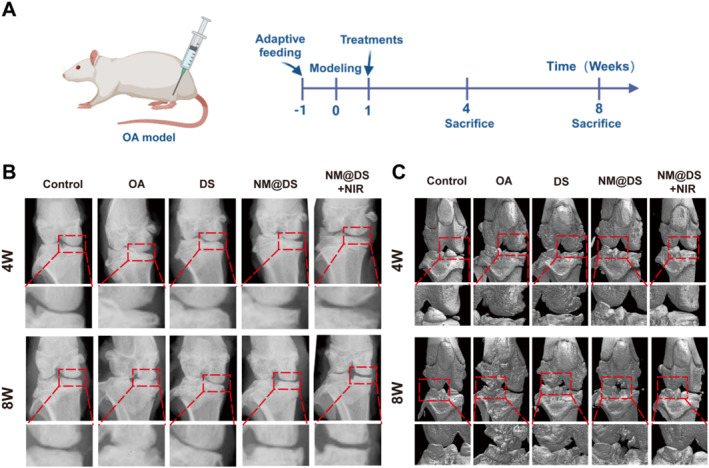
Evaluation of the therapeutic effect of the microsphere hydrogel system in a rat OA model by X‐ray and micro‐CT. (A) Schematic representation of the animal experiments used in this work. (B) Representative X‐ray images of knee joints from rats in the different groups after 4 and 8 weeks of treatment. (C) Representative magnetic resonance imaging images of knee joints from rats in different groups after 4 and 8 weeks of treatment. (*n* = 5 in each group).

Next, a rat osteoarthritis animal model was established using the destabilization of the medial meniscus (DMM) method (Figure S6), a well‐established and widely used protocol for the construction of OA models with good reliability, reproducibility, and structural similarity to that of human beings [48, 49]. 7 days post surgery, the rats were treated with DS and DS‐encapsulated microspheres, with or without NIR irradiation, for 4 or 8 weeks, and their therapeutic effects were evaluated using X‐ray, CT, and tissue staining (Figure 5A).

X‐ray results demonstrated that the joint space in the OA group was narrower at 4 weeks post‐surgery than that in the sham group and worsened at 8 weeks post‐surgery. Osteophytes were observed in the OA group, suggesting a well‐established OA rat model. However, following treatment with DS and DS‐encapsulated microspheres alone or in combination with NIR irradiation, the joint space was restored compared with that in the OA group, exhibiting a reduction in osteophyte formation (Figure 5B). Notably, treatment with DS‐encapsulated microspheres with NIR irradiation exhibited the most distinct joint spaces among the three treatment groups, suggesting that the combination of NIR irradiation‐based photothermal response and enhanced lubrication provides superior therapeutic benefits over simply increasing lubrication and slow drug release using microspheres alone. Furthermore, micro‐CT images of the knee joints from each group were also analyzed to show reduced osteophytes, restored joint space, and smoother joint surfaces in the three treatment groups compared with the OA group, exhibiting the optimal outcome in the DS‐encapsulated microspheres with NIR irradiation group (Figure 5C).

**FIGURE 6:**
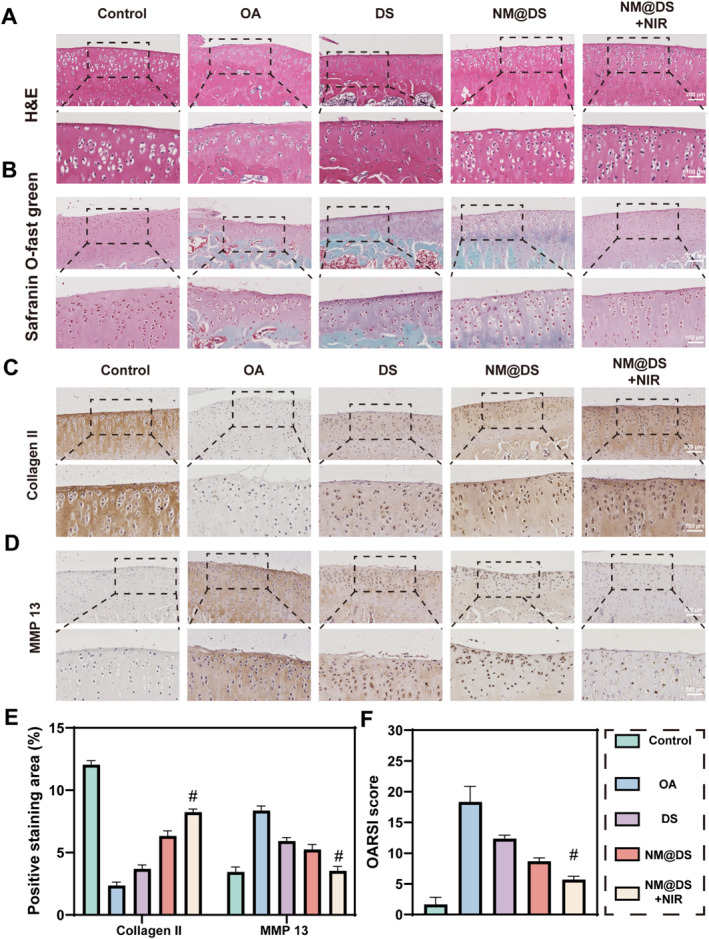
Histological and OARSI evaluation of the knee joints of rats after 8‐week treatment in the different groups. (A, B) Representative images of hematoxylin and eosin (H & E), safranin O, and fast green (S & O) staining of knee joints from rats after 8‐week treatment in different groups. (C–E) Representative images of immunohistochemical staining for collagen II (C), MMP ‐13 (D), and quantitative analysis (E) for knee joints from rats after 8‐week treatment in different groups. (F) Quantitative analysis of Osteoarthritis Research Society International (OARSI) grades. (*n* = 5, #*p* < 0.0001).

Histopathological analysis was further performed using H&E staining to reveal that the disruption of tightly packed chondrocytes, reduced cartilage thickness, abnormal chondrocyte morphology, and erosion cracks were observed in the OA group. However, these degenerative changes were significantly alleviated in the three treatment groups by increasing the cartilage thickness and suppressing chondrocyte apoptosis, particularly in the DS‐encapsulated microspheres with NIR irradiation group (Figure 6A). Safranin O‐Fast Green (S‐O) staining showed improved superficial/deep GAG red staining and decreased calcified and subchondral bone tissue green staining after 8 weeks of treatment with DS‐encapsulated microspheres with NIR irradiation compared to the OA group. This indicated that DS‐encapsulated microspheres as intelligent drug carriers and biolubricants at the joint interface provided superior therapeutic effects for OA (Figure 6B).

The protein levels of collagen II and MMP 13 were also evaluated using immunohistochemical staining, and the positive intensity of collagen II was lowest in the OA group, as shown in Figure 6C,E, which was restored after treatment with DS and DS‐encapsulated microspheres, with or without NIR irradiation, and was highest in the DS‐encapsulated microspheres with NIR irradiation group. Conversely, MMP‐13 protein expression (Figure 6D,E) showed an inverse pattern, being the highest in the OA group, downregulated among the three groups, and the lowest in the DS‐encapsulated microspheres with NIR irradiation group. A complementary analysis of inflammatory mediators further supported this regulatory pattern as TNF‐α expression followed a similar trend (Figure S7). Furthermore, the evaluation of cartilage damage by the Osteoarthritis Research Society International (OARSI) scoring system in the DS‐encapsulated microspheres with NIR irradiation group also exhibited a lower score than that of the OA group, and the lowest in the three treatment groups, suggesting a superior therapeutic effect of DS‐encapsulated microspheres with NIR irradiation treatment (Figure 6F). These results suggested that DS‐encapsulated microspheres with NIR irradiation can improve collagen II degradation and downregulate MMP‐13 expression, potentially contributing to cartilage damage repair.

Taken together, these results indicate that intra‐articular injections of DS‐encapsulated microspheres effectively lubricated the joints and slowed the progression of OA. Owing to the short half‐life of DS, its anti‐inflammatory effects rapidly diminish after direct intra‐articular injection, which requires frequent administration to maintain efficacy. As a result, direct intra‐articular injection of DS provides limited relief from osteoarthritis. However, the DS‐encapsulated microspheres improved the retention of the DS in the joint cavity, enabling efficient drug release under near‐infrared laser irradiation, thereby prolonging the anti‐inflammatory effect of the drug. In addition, the lubricating properties of the DS‐encapsulated microspheres contributed to a synergistic anti‐inflammatory and lubricating effect in OA treatment.

## 4 Conclusion

In this study, we prepared MXene/NIPIAM‐based photothermal‐responsive injectable hydrogel microspheres using a microfluidic system with a uniform size and excellent lubrication performance, which could be used to intervene in the physical microenvironment of lubrication impairment in OA. In addition, the prepared hydrogel microspheres, characterized by a solid photothermal response under NIR irradiation, were loaded with an anti‐inflammatory drug, diclofenac sodium, and exhibited attractive controlled release of agents, thus demonstrating beneficial therapeutic effects in the treatment of OA by modulating the physical lubrication and chronic chemical inflammation microenvironment, which is important for OA treatment and regenerative medicine.

## Author Contributions

**Zehua Gong:** conceptualization, methodology, software, investigation, formal analysis, writing – original draft. **Linjie Chen:** data curation, investigation, formal analysis, writing – original draft. **Xiaolei Zhou:** visualization, investigation. **Chunwu Zhang:** formal analysis, writing – review and editing. **Dražen Matičić:** formal analysis, writing – review and editing. **Dražen Vnuk:** formal analysis, writing – review and editing. **Zhifeng You:** formal analysis, writing‐ original draft, writing – review and editing. **Linjin Li:** funding acquisition, writing – review and editing. **Huaqiong Li:** conceptualization, funding acquisition, resources, supervision, writing – review and editing. All authors reviewed the manuscript.

## Ethics Statement

The animal experiments in this study were approved by the Laboratory Animal Ethics Committee of Wenzhou Institute, University of Chinese Academy of Sciences.

## Conflicts of Interest

The authors declare no conflicts of interest.

## Supporting information

Supporting Information S1

## Data Availability

The data that support the findings in this study are available from the last corresponding author upon reasonable request.
