# Supplementary material for: MXene‐Based Photothermal‐Responsive Injectable Hydrogel Microsphere Modulates Physicochemical Microenvironment to Alleviate Osteoarthritis
Source: Smart Med. 2025 Apr 13;4(2):e70006. doi: 10.1002/smmd.70006 (PMC11994158; doi:10.1002/smmd.70006)
Supplement: Supplementary file 1 — Supporting Information S1 [file SMMD-4-e70006-s001.docx]

Supporting Information

Mxene-based photothermal-responsive injectable hydrogel microsphere modulates physicochemical microenvironment to alleviate osteoarthritis

Zehua Gong^1,2,8#^, Linjie Chen^3#^, Xiaolei Zhou^4^, Chunwu Zhang^5^, Dražen Matičić^6^, Dražen Vnuk^6^, Zhifeng You^2^*, Linjin Li^7^* and Huaqiong Li^1,2^*

^1^Joint Research Centre on Medicine, Xiangshan Hospital of Wenzhou Medical University, Ningbo, Zhejiang 315700, P. R. China

^2^Zhejiang Engineering Research Center for Tissue Repair Materials, Wenzhou Institute, University of Chinese Academy of Sciences, Wenzhou, Zhejiang 325001, P. R. China

^3^Department of Orthopaedics, Key Laboratory of Orthopaedics of Zhejiang Province, The Second Affiliated Hospital and Yuying Children's Hospital of Wenzhou Medical University, Wenzhou, Zhejiang 325035, P. R. China

^4^Jiangxi Provincial Key Laboratory of Tissue Engineering, School of Rehabilitation Medicine, Gannan Medical University, Ganzhou, Jiangxi 341000, P. R. China

^5^Joint Centre of Translational Medicine, The First Affiliated Hospital of Wenzhou Medical University, Wenzhou, Zhejiang 325035, P. R. China

^6^Clinic for Surgery, Orthopaedics and ophthalmology, Faculty of Veterinary Medicine, University of Zagreb, Heinzelova 55, 10000 Zagreb, Croatia

^7^Department of Urology, The Third Clinical Institute Affiliated to Wenzhou Medical University,Wenzhou People's Hospital, Wenzhou, Zhejiang 325000, China

^8^The Fifth Hospital of Jinhua, Jinhua, Zhejiang 321000, P. R. China

*Correspondence to: [zfyou2020@126.com](mailto:zfyou2020@126.com), [157173613@qq.com](mailto:157173613@qq.com), [lihq@ucas.ac.cn](mailto:lihq@ucas.ac.cn)

# contributed equally to this work

This file includes:

Figure S1 to S7

Table S1 to S2

**Table S1.** RNA primer sequence

| **Gene** | **Forward sequence（5’-3’）** | **Reverse sequence（3’-5'）** |
| --- | --- | --- |
| Aggrecan | GATCTCAGGGGGGG | TCCACAAACGTAATGCCAGA |
| Collagen II | CTCAAGTCGCTGAACAACCA | GTCTCCGCTTCCACTG |
| MMP 13 | AACCAAGATGTGGAGTGCCTGATG | CACATCAGACCAGACCTTGAAGGC |
| ADAMTS 5 | TCCTCTTGGTGGCTGACTCTTCC | TGGTTCTCGATGCTTGCATGACTG |
| GAPDH | GAAGGTCGGTGTGAACGGATTTG | CATGTAGACCATGTAGTTGAGGTCA |

**Table S2.** Reference range for ALT, AST, BUN and CRE in healthy rat serum

| **Indicator** | **Reference range** |
| --- | --- |
| ALT (U/L) | 50.55-86.56 |
| AST (U/L) | 129.55-237.34 |
| BUN (mmol/L) | 2.15-7.41 |
| CRE (μmol/L) | 50.88-75.90 |

**
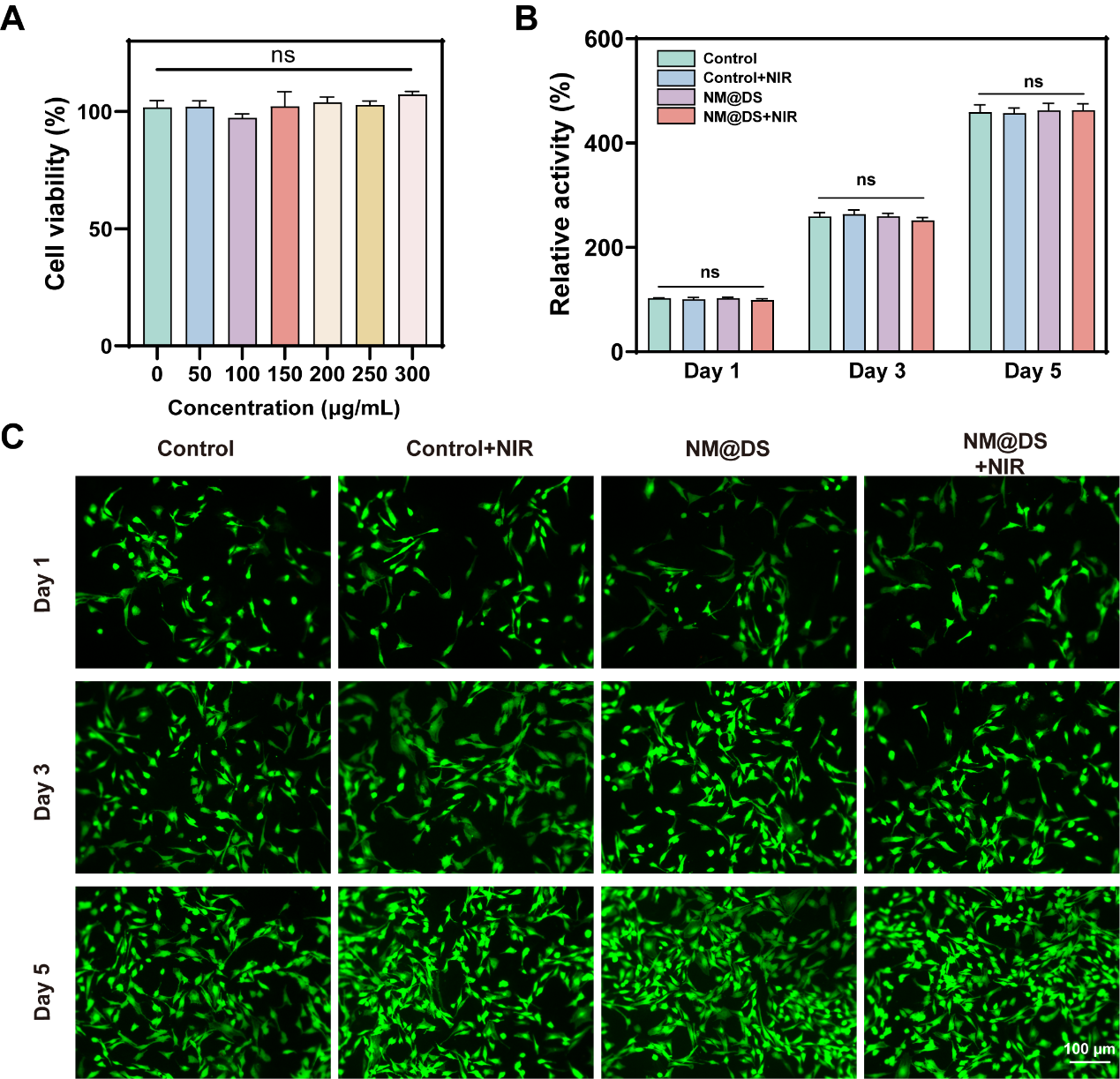
**

**Figure S1.** Biocompatibility evaluation of the hydrogel microspheres with primary chondrocytes. **A)** Relative viability of primary chondrocytes treated with extracts from microsphere hydrogels with different concentrations of MXene ranging from 0 to 300 μg/mL for 24 h (n=6 per group). **B)** Relative viability of primary chondrocytes treated with extracts from microsphere hydrogels with or without diclofenac sodium (DS) under near-infrared (NIR) irradiation for 1, 3, and 5 days (n=6 per group). C) Representative images of primary chondrocytes cultured with extracts from microsphere hydrogels or treated with NIR and stained using a live-dead staining kit. Scale Bar, 100 μm.


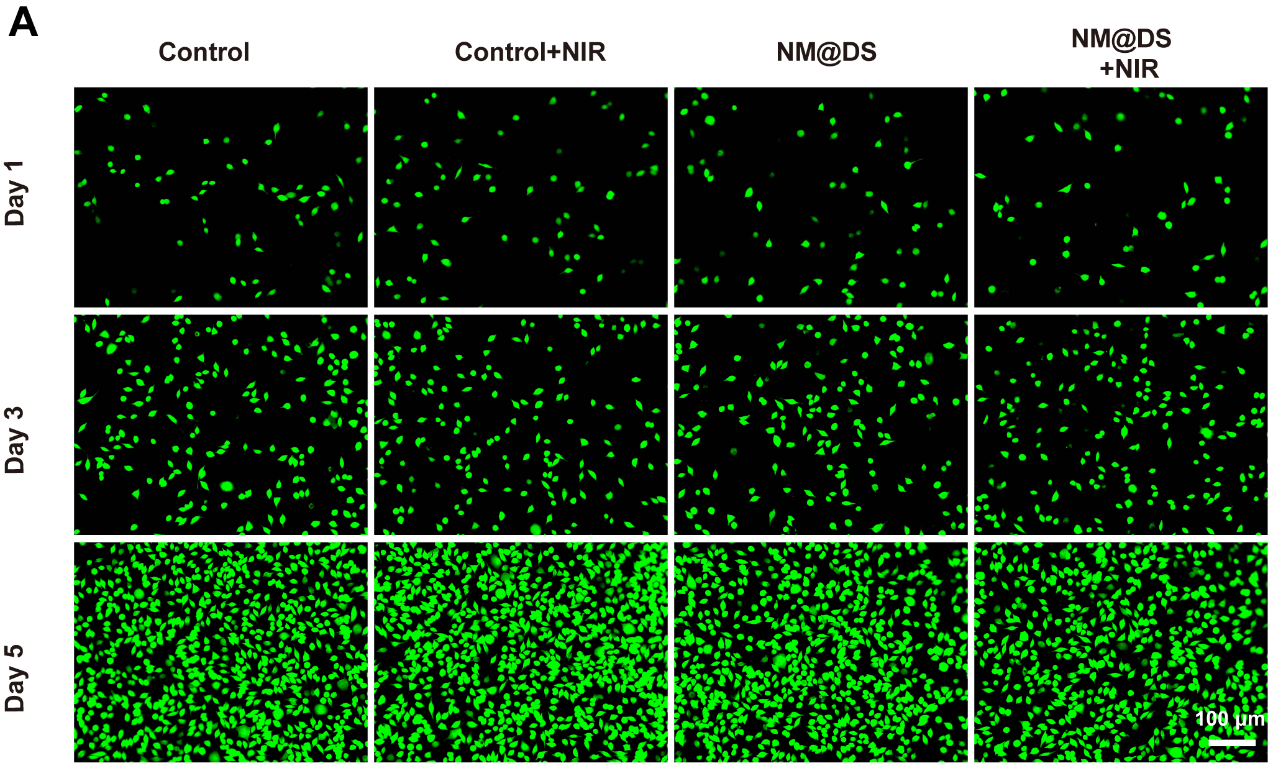


**Figure S2.** Biocompatibility evaluation of the hydrogel microspheres with L929 cells. **A)** Live/dead staining images of L929 cells cultured in extract treated with extracts from microsphere hydrogels with or without diclofenac sodium (DS) under near-infrared (NIR) irradiation for 1, 3, and 5 days.Scale bar, 100 μm.


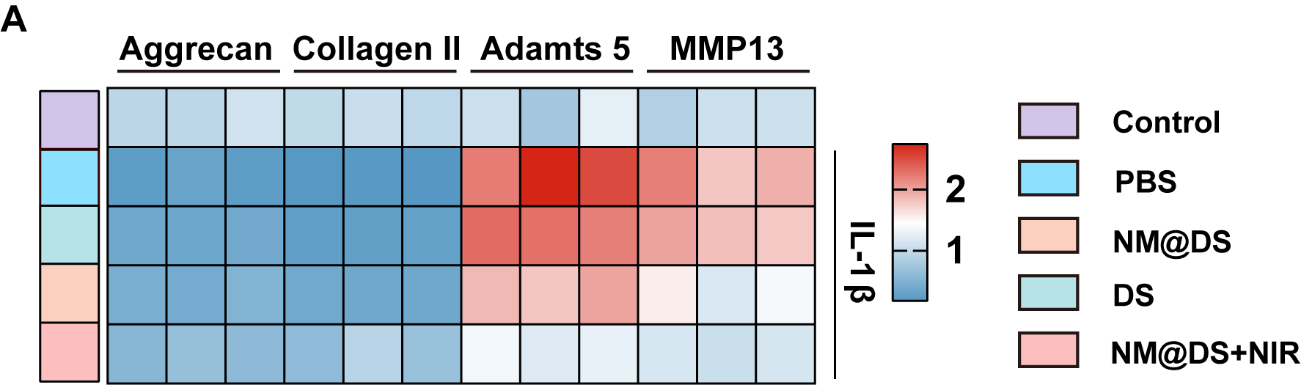


**Figure S3.** Beneficial effects of the NIR-responsive release of DS-loaded microsphere hydrogels on chondrocytes.**(A)**Relative gene expression of Aggrecan, Collagen II, ADAMTS 5, MMP 13 in chondrocytes from each group.

**
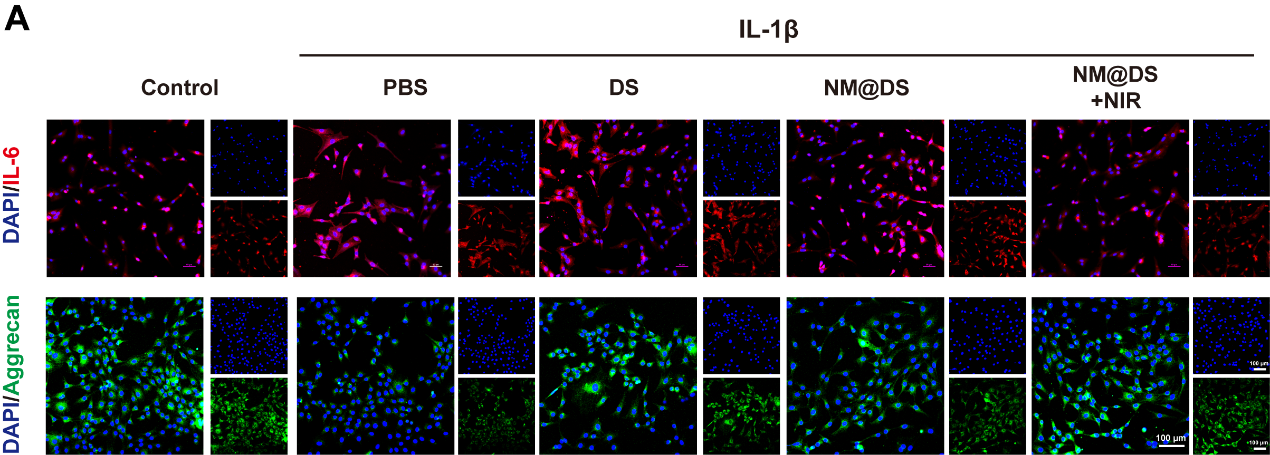
**

**Figure S4.** Beneficial effects of the NIR-responsive release of DS-loaded hydrogel microsphere on chondrocytes. **A)** Representative immunofluorescence images of IL-6 (red) and Aggrecan (green) in chondrocytes from different groups. Scale Bar, 100 μm.

**
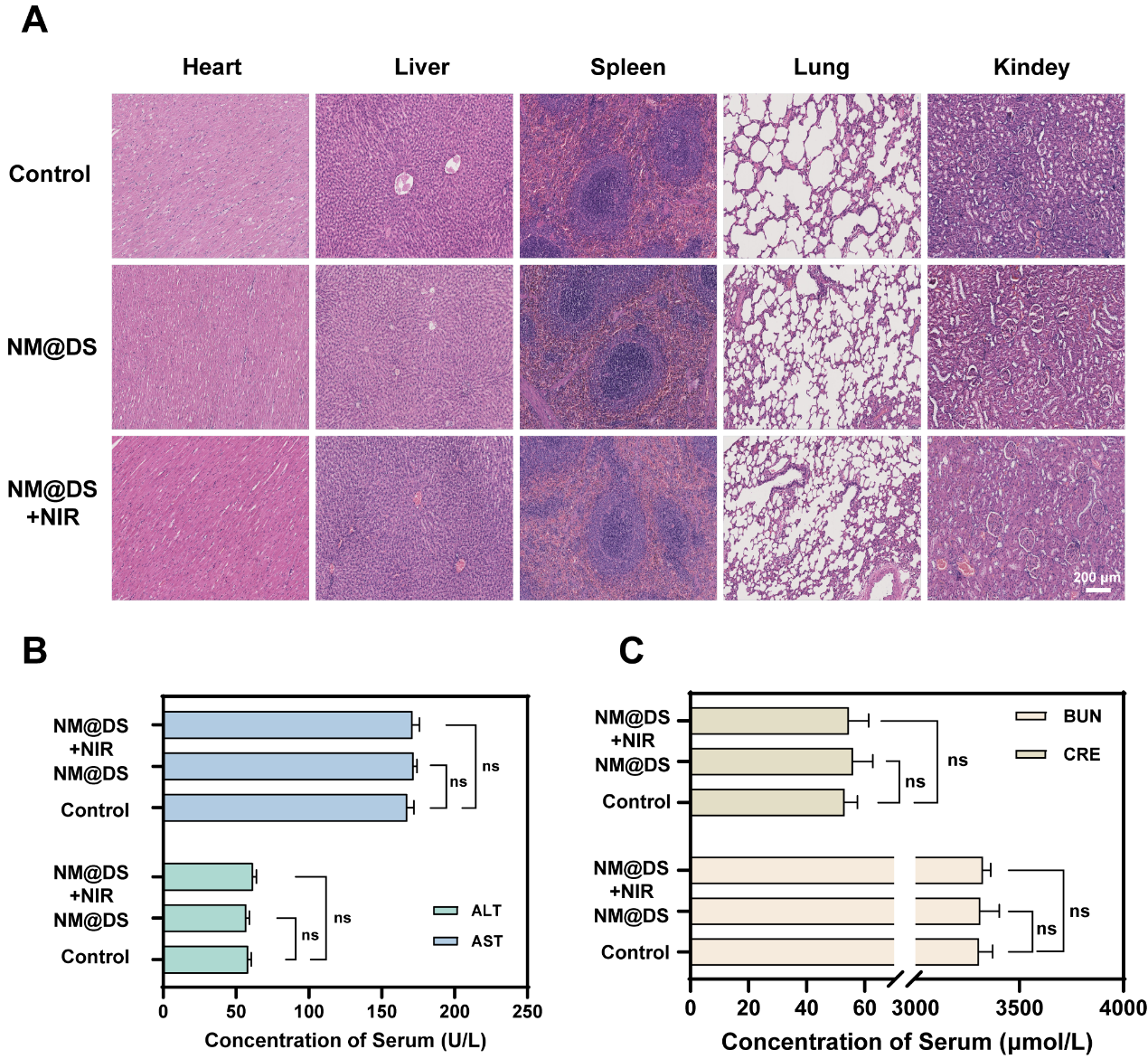
**

**Figure S5.** Evaluation of the in vivo biocompatibility of hydrogel microspheres. **A)** Hematoxylin-eosin (H&E) staining of the heart, liver, spleen, lungs, and kidneys from each group of rats after 8 weeks of treatment. **B)** Serum ALT and AST levels in each group of rats after 8 weeks of treatment. **C)** Serum CRE and BUN levels in each group of rats after 8 weeks of treatment. (n=5, Scale bar, 200μm).


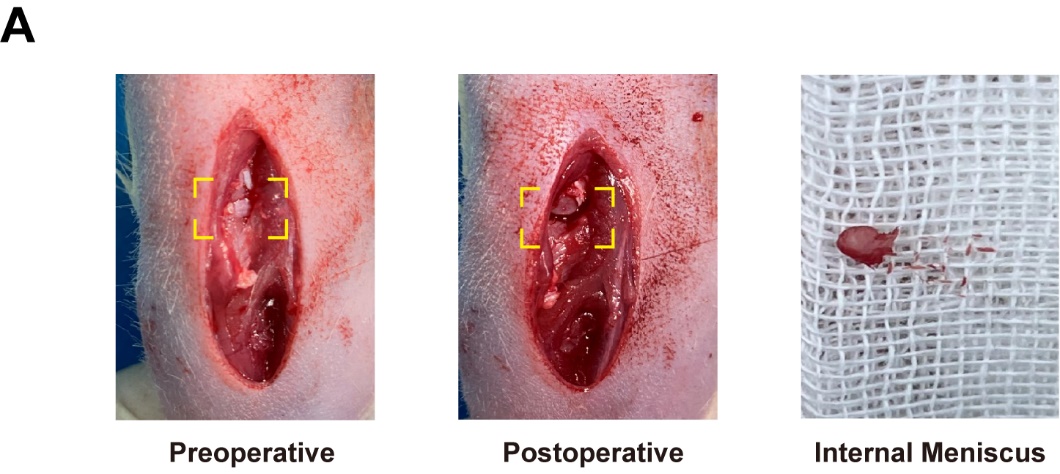


**Figure S6.** DMM surgical removal of the medial meniscus.


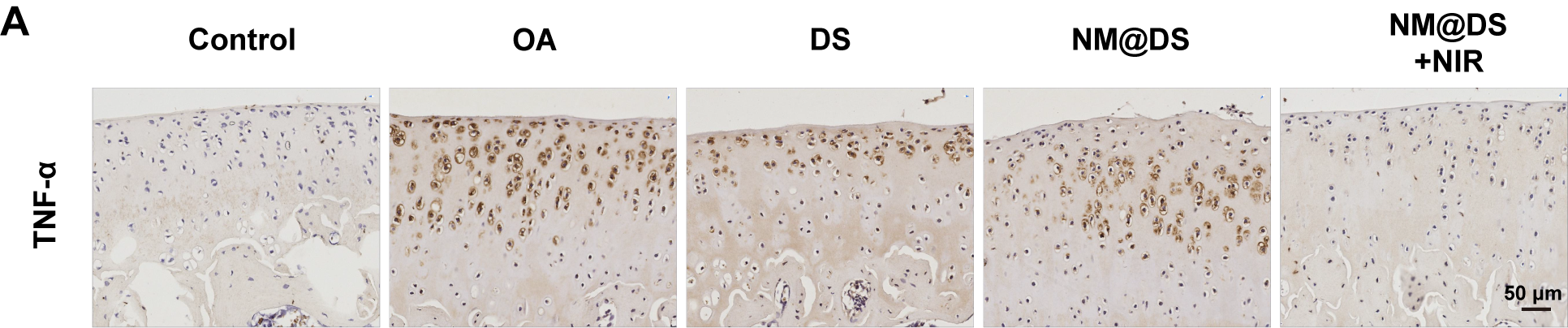


**Figure S7**. Immunohistochemistry of the knee joint. **A)** Immunohistochemical staining of TNF-α on articular surface of cartilage, Scale Bar: 50 μm.
